# Supplementary material for: Resolving the full spectrum of human genome variation using Linked-Reads
Source: Genome Res. 2019 Apr;29(4):635–45. doi: 10.1101/gr.234443.118 (PMC6442396; doi:10.1101/gr.234443.118)
Supplement: Supplemental Material [file supp_gr.234443.118_Supplemental_longranger-2.2.zip › longranger-2.2.tar.bz2!/lr2.2-public/lib/stan/cmdstan/stan/doc/api/html/structstan_1_1agrad_1_1_operands_and_partials-members.html]

Stan: Member List


|  |  |
| --- | --- |
|  | Stan  2.5.0  probability, sampling & optimization |


- Main Page
- Related Pages
- Namespaces
- Classes
- Files

- Class List
- Class Index
- Class Hierarchy
- Class Members

All Classes Namespaces Files Functions Variables Typedefs Enumerations Enumerator Friends Macros Pages

- stan
- agrad
- OperandsAndPartials

stan::agrad::OperandsAndPartials< T1, T2, T3, T4, T5, T6, T\_return\_type > Member List

This is the complete list of members for stan::agrad::OperandsAndPartials< T1, T2, T3, T4, T5, T6, T\_return\_type >, including all inherited members.

|  |  |  |
| --- | --- | --- |
| all\_constant | stan::agrad::OperandsAndPartials< T1, T2, T3, T4, T5, T6, T\_return\_type > | static |
| all\_partials | stan::agrad::OperandsAndPartials< T1, T2, T3, T4, T5, T6, T\_return\_type > |  |
| all\_varis | stan::agrad::OperandsAndPartials< T1, T2, T3, T4, T5, T6, T\_return\_type > |  |
| d\_x1 | stan::agrad::OperandsAndPartials< T1, T2, T3, T4, T5, T6, T\_return\_type > |  |
| d\_x2 | stan::agrad::OperandsAndPartials< T1, T2, T3, T4, T5, T6, T\_return\_type > |  |
| d\_x3 | stan::agrad::OperandsAndPartials< T1, T2, T3, T4, T5, T6, T\_return\_type > |  |
| d\_x4 | stan::agrad::OperandsAndPartials< T1, T2, T3, T4, T5, T6, T\_return\_type > |  |
| d\_x5 | stan::agrad::OperandsAndPartials< T1, T2, T3, T4, T5, T6, T\_return\_type > |  |
| d\_x6 | stan::agrad::OperandsAndPartials< T1, T2, T3, T4, T5, T6, T\_return\_type > |  |
| nvaris | stan::agrad::OperandsAndPartials< T1, T2, T3, T4, T5, T6, T\_return\_type > |  |
| OperandsAndPartials(const T1 &x1=0, const T2 &x2=0, const T3 &x3=0, const T4 &x4=0, const T5 &x5=0, const T6 &x6=0) | stan::agrad::OperandsAndPartials< T1, T2, T3, T4, T5, T6, T\_return\_type > | inline |
| to\_var(double logp) | stan::agrad::OperandsAndPartials< T1, T2, T3, T4, T5, T6, T\_return\_type > | inline |


---

[ Stan Home Page ]

*© 2011–2014,
Stan Development Team.*
